# A Case of Pediatric Internal Hernia Heralded by Constipation

**DOI:** 10.7759/cureus.75185

**Published:** 2024-12-05

**Authors:** Clates P Adams, Linda Y Meyers, Ricardo J Rodriguez, Howard O Neil, Joshua J Oliver

**Affiliations:** 1 Emergency Medicine, Madigan Army Medical Center, Tacoma, USA; 2 General Surgery, Bassett Army Hospital, Fort Wainwright, USA

**Keywords:** abdominal pain, case report, constipation, emergency medicine, internal hernia, pediatrics, pediatric surgery

## Abstract

Meckel’s diverticulum (MD) is the most common gastrointestinal congenital anomaly of the small intestine. A small subset of patients with MD develops a mesodiverticular band (MDB), creating a snare-like opening and the potential for internal hernias (IHs). IHs are a known possible cause of small bowel obstructions and are most common in adults post bariatric surgery. Herein, we present an atypical case of pediatric internal hernia caused by an MDB. A six-year-old male child with chronic constipation presented with one week of abdominal pain and one day of non-bloody, non-bilious emesis, decreased appetite, and normal non-bloody bowel movements. The patient appeared uncomfortable with mild abdominal right upper quadrant tenderness. Laboratory results were remarkable for mild leukocytosis and hyperkalemia. Computed tomography was concerning for IH. General surgery performed a diagnostic laparotomy, revealing an IH caused by an MDB. The MDB was released, and the patient had an uneventful recovery. Pediatric IHs are very rare. Surgery represents the primary management of symptomatic MDBs, as it allows for the hernia to be released before complications occur, such as bowel necrosis or gangrene. This case highlights the importance of considering IH caused by MDB in pediatric patients presenting with constipation.

## Introduction

Meckel’s diverticulum (MD) is the most common gastrointestinal congenital anomaly of the small intestine, occurring in roughly 2% of the world’s population, of which only 2% of cases become symptomatic [1,2]. MD traditionally arises within two feet of the ileocecal valve [3], originating from an incomplete obliteration of the vitelline duct during gestation [1]. Approximately 5% of all patients with MD develop a mesodiverticular band (MDB) [4]. The incidence of MDB is three times higher in males than in females, with no age predilection [1].

MDB can create a snare-like opening through which bowel loops may internally herniate. This process can cause incarceration or strangulation of the hernia along with hemorrhage secondary to traumatic rupture of the MBD [1]. Patients classically present with painless rectal bleeding, with some children having complaints of abdominal cramping, vomiting, and abdominal distension. However, patients can also present asymptomatically or with intermittent colicky pain [1,3,4]. A Meckel’s scan (technetium-99m) is the diagnostic modality of choice, and a definitive diagnosis is made with laparoscopy or laparotomy [5].

Internal hernias (IHs) are responsible for 0.6-5.8% of all small bowel obstructions, leading to significant morbidity and mortality reaching 50% if strangulated and untreated [5,6]. Herein, we present a unique case of a pediatric IH in a pediatric patient who had none of the risk factors listed above.

## Case presentation

A six-year-old male child presented with his mother to our department with one week of midline abdominal pain, one day of non-bloody, non-bilious emesis, and decreased appetite, and a normal, non-bloody bowel movement the day before. The patient had a history of chronic constipation requiring polyethylene glycol 3350 and docusate capsules daily, for which he had been admitted previously.

**Figure 1:**
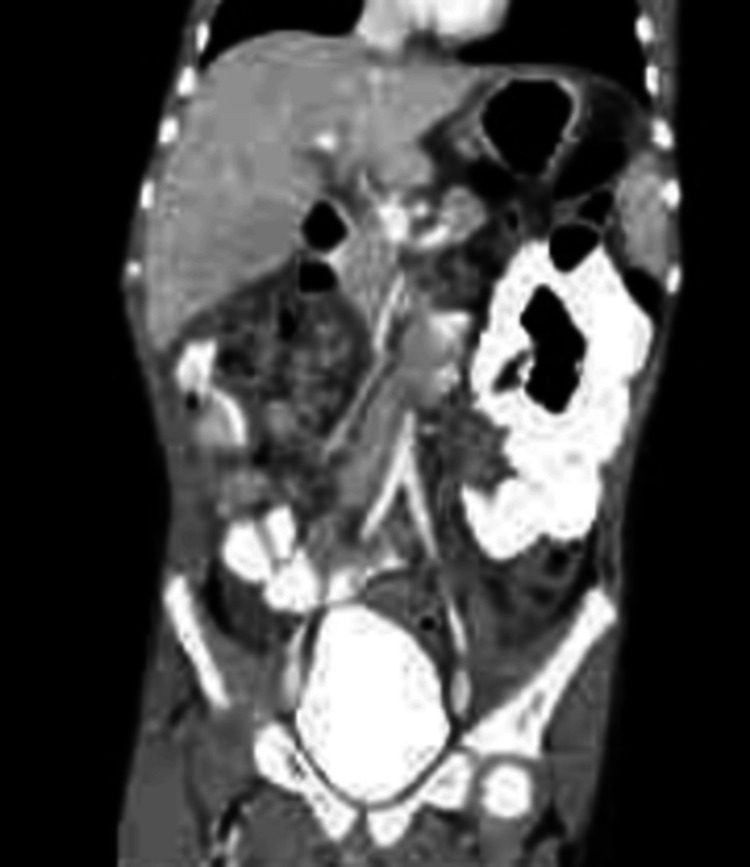
Computed tomography (CT) with oral and IV contrast demonstrating dilated loops of bowel

On physical examination, the patient’s vital signs were unremarkable. He appeared uncomfortable but not toxic, fatigued, with mild abdominal right upper quadrant tenderness and diffuse, hyperactive bowel sounds. Workup and resuscitation were initiated to address the mother’s concerns, comprising an intravenous (IV) fluid bolus and IV ondansetron, which initially improved the patient’s symptoms. The workup included a complete blood count (CBC), complete metabolic panel (CMP), lipase, point-of-care glucose, urine analysis, and computed tomography (CT) abdomen and pelvis without contrast. His CBC and CMP demonstrated leukocytosis and hyperkalemia, respectively, with the remainder of his laboratory evaluation being unremarkable, as seen in Tables 1-3. Initial CT showed signs of mesenteric adenitis and colitis, and dilated loops of bowel, indicating a possible IH, as seen in Figure 1.

**Table 1:** Complete blood count (CBC) results

Parameter	Patient Value	Reference Value
White blood cells	11.28 × 10^3^/uL	4.5–11.0 × 10^3^/uL
Red blood cells	5.38 × 10^6^/uL	4.0–5.20 × 10^6^/uL
Hemoglobin	14.70 g/dL	10.0–15.0 g/dL
Hematocrit	41.8%	34%–45%
Mean corpuscular volume	77.7 fL	80–98 fL
Mean corpuscular hemoglobin	27.3 pg	26.7–33.7 pg
Mean corpuscular hemoglobin concentration	35.2 g/dL	32.5–37.5 g/dL
Red cell distribution width	12.4%	11.5%–15.0 %
Platelets	415 × 10^3^/uL	140–420 × 10^3^ uL
Mean platelet volume	9.5 fL	7.0–12.0 fL
Neutrophil %	78.4%	38.5%–76.5%
Lymphocyte %	15.7%	14.0%–46.0%
Monocyte %	5.0%	3.0%–13.0%
Eosinophil %	0.1%	0.0%–7.4%
Basophil %	0.4%	0.0%–2.5%
Immature granulocyte %	0.4%	0.0 – 2.0%
Neutrophil absolute	8.86 × 10^3^/uL	1.50–10.0 × 10^3^/uL
Lymph absolute	1.77 × 10^3^/uL	0.90–3.00 × 10^3^/uL
Mono absolute	0.56 × 10^3^/uL	0.20–0.90 × 10^3^/uL
Eosinophil absolute	<0.03 × 10^3^/uL	0.0–0.40 × 10^3^/uL
Basophil absolute	0.04 × 10^3^/uL	0.0–0.20 × 10^3^/uL
Nucleated red blood cell %	0.00%	0.0%–5.0%
Nucleated red blood cell Absolute	0.00 × 10^3^/uL	0.00–2.0 10^3^/uL
Immature granulocyte absolute	0.04 × 10^3^/uL	0.0–2.0 10^3^/uL

**Table 2:** Comprehensive metabolic panel (CMP) Results BUN: blood urea nitrogen

Parameter	Patient Value	Reference Value
Sodium	136 mmol/L	135–145 mmol/L
Potassium	4.53 mmol/L	3.50–5.10 mmol/L
Chloride	99 mmol/L	98–107 mmol/L
Carbon dioxide	20 mmol/L	22–31 mmol/L
Anion gap	17 mmol/L	7–16 mmol/L
Osmo Calc	271 mOsm/kg	277–308 mOsm/kg
BUN	8.5 mg/dL	6.0–23.0 mg/dL
Creatine Level	0.35 mg/dL	0.50–1.0 mg/dL
BUN/Creatine Ratio	24.3	7–25
Glucose	100 mg/dL	74–109 mg/dL
Calcium	9.6 mg/dL	8.6–10.3 mg/dL
Protein Total	7.3 g/dL	6.6–8.7 g/dL
Albumin	4.6 g/dL	3.5–5.2 g/dL
Bilirubin Total	0.34 mg/dL	0.15–1.20 mg/dL
Alkaline phosphatase	164 U/L	35–104 U/L
Alanine transaminase	7 U/L	0–33 U/L
Aspartate aminotransferase	21 U/L	0–35 U/L
Lipase	9 U/L	13–60 U/L

**Table 3:** Urine analysis (UA) results

Parameter	Patient Value	Reference Value
Color	Yellow	Colorless – High
Appearance	Clear	Clear – High
pH	7	≥0 – High
Specific Gravity	>1.035	1.003–1.035
UA Glucose	Negative	Negative – High
UA Ketones	80	Negative – High
UA Blood	Negative	Negative – High
UA Protein	Negative	Negative – High
UA Bilirubin	Negative	Negative – High
UA Urobilinogen	<2.0	<2.0 – High
UA Nitrate	Negative	Negative – High
UA Leukocyte esterase	Negative	Negative – High
UA White Blood Cells	<1 / HPF	0 – ≤3 / HPF
UA Red Blood Cells	2 / HPF	0–3 /HPF
UA Squamous epithelial cells	<1 / HPF	0 – ≤10 /HPF

General Surgery was consulted and on initial exam found a non-surgical abdomen. However, soon after evaluation, the patient’s pain worsened and a second bolus of IV fluids was given along with IV morphine. A repeat CT abdomen and pelvis with oral and IV contrast were ordered, demonstrating proximal to mid-small bowel distention and an abrupt transition of decompressed small bowel likely representing small bowel obstruction caused by a suspected internal hernia. General Surgery was re-consulted, and the patient was taken for a diagnostic laparotomy.

**Figure 2:**
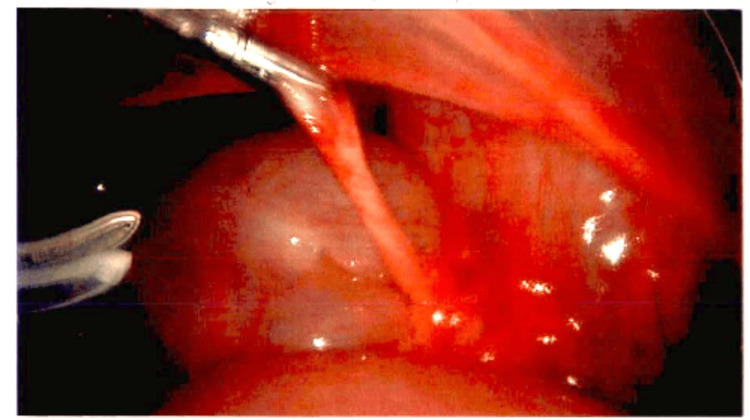
Intraoperative image showing snare-like mesodiverticular band

A band-like structure was seen entrapping the small bowel, as seen in Figure 2. Pathologic analysis revealed fibroadipose tissue with fibroblastic proliferation, mesothelial hyperplasia, and dilated blood vessels consistent with MDB. On follow-up, the patient was stable without residual abdominal pain and was tolerating oral intake without complications. 

## Discussion

Pediatric IHs are very rare, with an estimated incidence of less than 1% [5,6]; they are much more common in adults post bariatric surgery due to altered intestinal anatomy [7]. Surgery is the primary management of symptomatic MDBs, as it allows for the hernia to be released before complications occur, such as bowel necrosis or gangrene [8]. Unless magnetic resonance imaging is immediately available, a CT scan with oral and IV contrast is likely required in pediatric patients with suspected MDB [8].

This report is unique in that it describes a pediatric case of an IH caused by an MDB with a unique presentation. As opposed to bloody stools, this patient presented with recurrent episodes of constipation. Although many are reluctant to order CT imaging on pediatric patients, this case highlights that sometimes repeat imaging is needed if there is increased suspicion of surgical pathology.

## Conclusions

This case highlights the importance of considering an IH caused by MDB in pediatric patients presenting with constipation. Furthermore, in cases with a high suspicion of surgical pathology, repeated imaging and consultation may be necessary to address a potential surgical emergency.
